# Pathways to Clinical Training Opportunities for International Medical Students and IMGs: The California Experience

**DOI:** 10.5334/aogh.5020

**Published:** 2026-01-08

**Authors:** Margaret Akey, Brian Groves, James C. Hudspeth, Tracy L. Rabin, Sarah Cortez, Rebecca Silvers, Joseph Novotny, Fekir Negussie, Patti Orozco, Phuoc Le, Riya Sawhney, Nakul Raykar, Michelle Arteaga, John A. Davis Rodriguez, Maureen Curran, Susan Byekwaso, Faysal Saab, Norkamari Shakira Bandolin, Michael S. Lipnick

**Affiliations:** 1 School of Medicine at the University of California, San Francisco, in San Francisco, CA, USA; 2 International Students and Scholars Office at the University of California, San Francisco, in San Francisco, CA, USA; 3 Department of Medicine at Boston University School of Medicine in Boston, MA, USA; 4 Department of Internal Medicine at Yale School of Medicine in New Haven, CT, USA; 5 Human Resources in the Department of Anesthesia at the University of California, San Francisco, in San Francisco, CA, USA; 6 Institute of Global Health Sciences at the University of California, San Francisco, in San Francisco, CA, USA; 7 Office of Research at the University of California, San Francisco, in San Francisco, CA, USA; 8 Institute of Global Health Sciences, Center for Health Equity in Surgery and Anesthesia at the University of California, San Francisco, in San Francisco, CA, USA; 9 Medical Education at Vin University College of Health Sciences in Hanoi, Vietnam; 10 Program in Global Surgery and Social Change in the Department of Global Health and Social Medicine at Harvard University in Boston, MA, USA; 11 Program in Global Surgery and Social Change in the Department of Global Health and Social Medicine at Brigham and Women’s Hospital, Harvard University, in Boston, MA, USA; 12 Global Engagement Programs Manager in the Department of Anesthesiology, Perioperative & Pain Medicine, Division of Global Health Equity at Stanford University School of Medicine in Stanford, CA, USA; 13 Medicine and Vice Chancellor of Education & Student Affairs at the University of California, San Francisco, in San Francisco, California, USA; 14 Administration and Business Development in Health Sciences International at the University of California, San Diego, in La Jolla, CA, USA; 15 Makerere University, College of Health Sciences in Kampala, Uganda; 16 Medicine and Pediatrics in the Hospitalist Section at the University of California, Los Angeles, in Los Angeles, CA, USA; 17 Emergency Department at the University of California, Davis, in Sacramento, CA, USA; 18 Department of Anesthesia at the University of California, San Francisco, in San Francisco, CA, USA

**Keywords:** global health education, clinical training, bidirectional exchange, international medical partnerships, California academic medical centers, medical education, equity, international medical students, international medical graduates

## Abstract

Many global health initiatives involve partnerships between US academic institutions and low- or middle-income country (LMIC) institutions, but substantial inequities exist in short-term clinical education exchange opportunities for LMIC institutions. US medical students and physicians frequently participate in clinical experiences abroad; however, equivalent opportunities in the US for LMIC medical students and international medical graduates (IMGs) are limited, inconsistent across states, and, for IMGs, typically restricted to observerships.

The authors aimed to identify pathways that facilitate clinical training and educational exchanges between California academic medical centers (CA AMCs) and LMIC institutions, and to explore the barriers and enablers for international medical students and IMGs to engage in clinical training in California. The authors conducted 16 semi-structured interviews with global health education stakeholders at CA AMCs and performed a desk review using PubMed, gray literature, and resources from the US State Department, Medical Board of California, and CA AMC websites to ensure the accuracy of information presented.

Key institutional challenges include liability concerns, limited program capacity, and funding constraints; additional barriers specific to IMGs include restrictive visa policies and medical board regulations. Enablers included innovative funding mechanisms, existing administrative infrastructure, and, for IMGs, familiarity with visa processes, and the use of the Medical Board of California’s Special Permits to enable participation in hands-on patient care.

Several potential approaches emerged to reduce barriers and support hosting international medical students and IMGs in hands-on clinical roles at CA AMCs. While these findings offer practical strategies for expanding such exchanges within California, they also highlight the need for broader policy changes, including advocacy for a new visa category dedicated to short-term clinical training exchanges.

This article advances the discourse on decolonizing global health by identifying mechanisms to allow equitable, bidirectional clinical training opportunities.

## Introduction

Global health initiatives frequently involve partnerships between US academic institutions and institutions in low- and middle-income countries (LMICs). However, these partnerships have well-documented inequities [1–4]. While US medical students and graduates frequently engage in short-term, hands-on clinical training, research, and cultural immersion abroad, LMIC counterparts rarely receive reciprocal opportunities in the US. Furthermore, although international medical students can engage in supervised, hands-on patient care during clinical electives at US institutions, international medical graduates (IMGs) who engage in short-term training are typically limited to clinical observerships [5]. This imbalance highlights a deficiency in US academic global health programs and a missed opportunity for improving global health equity [6, 7].

Legal and institutional challenges have previously been identified as barriers to IMGs gaining access to short-term, hands-on clinical training in the US [6, 8]. Such training equips participants to manage a broader range of scenarios, fosters the exchange of ideas, and strengthens local healthcare systems when trainees return home. US institutions also benefit from the perspectives and expertise of visiting trainees, creating pathways for sustained collaboration and research [7, 9].

In 2019, Hudspeth et al. outlined key reforms needed to support global physician training, including a federal call for a new J-1 visa category for short-term clinical exchange, more state-level temporary licensure options, and institutional memoranda of understanding with LMIC partners [6]. Some states have made progress—Tennessee and Ohio, for example, have created new pathways for IMGs to engage in supervised clinical training [10–12]. California, home to 45 Association of American Medical Colleges (AAMC) institutions, has significant potential to expand training programs if effective pathways are identified, yet no effort has examined opportunities and barriers in the state [13].

This article aims to identify strategies to improve equity in global health partnerships and increase training opportunities for international medical students and IMGs at California Academic Medical Centers (CA AMCs) by:

1. **Examining barriers and enablers** at federal, state, and institutional levels.
2. **Identifying existing pathways** that support clinical global health exchanges in California.
3. **Proposing recommendations** to expand programs and advocate for policy reforms.

From May to August 2024, we gathered information using semi-structured interviews with 16 stakeholders from CA AMCs and a desk review using PubMed, gray literature, and relevant regulatory and institutional resources. This process was determined to be exempt from Institutional Review Board review.

### Barriers preventing clinical training opportunities for international medical students and IMGs

Multiple barriers limit access to short-term exchange programs that bring international medical students and IMGs to California.

Regulatory and administrative barriers are common for IMGs—visa processes are lengthy, expensive, and complex, with many visa types prohibiting clinical interaction. Obtaining a California Medical License or Special Permit requires a Social Security Number (SSN) or Individual Taxpayer Identification Number (ITIN), fingerprinting with the DOJ and FBI, and other steps that are burdensome for short-term visitors. Federal issues, such as the 2025 temporary freeze on J-1 visa interviews and travel restrictions for certain countries, add further obstacles [14].

Financial barriers are also commonly encountered for both IMGs and students, including travel and housing costs, stipend considerations, and California’s high living expenses. Limited university housing forces reliance on costly rentals or hotels, and many LMIC institutions cannot fund their trainees. Lack of credit history or US identification further complicates financial support.

Institutional capacity constraints arise in settings already managing high volumes of local trainees, with limited spots prioritized for US enrollees. Hosting requires extensive paperwork, multi-departmental coordination, and navigating challenges such as language, transportation, and family needs.

Political shifts at institutional, state, or federal levels—through changes in leadership, priorities, or policy—create additional uncertainty. Many of these barriers resemble those encountered by US medical students and physicians abroad, yet greater institutional investment is typically made to overcome them. A similar commitment is needed to build truly equitable, bidirectional training opportunities in California. Recent federal actions, such as steep new H-1B visa fees and restrictions, further highlight how fragile visa access for international medical training programs can be, underscoring the urgency of coordinated state and institutional responses [15–17].

### Enablers facilitating clinical training opportunities for international medical students and IMGs

**Figure 1:**
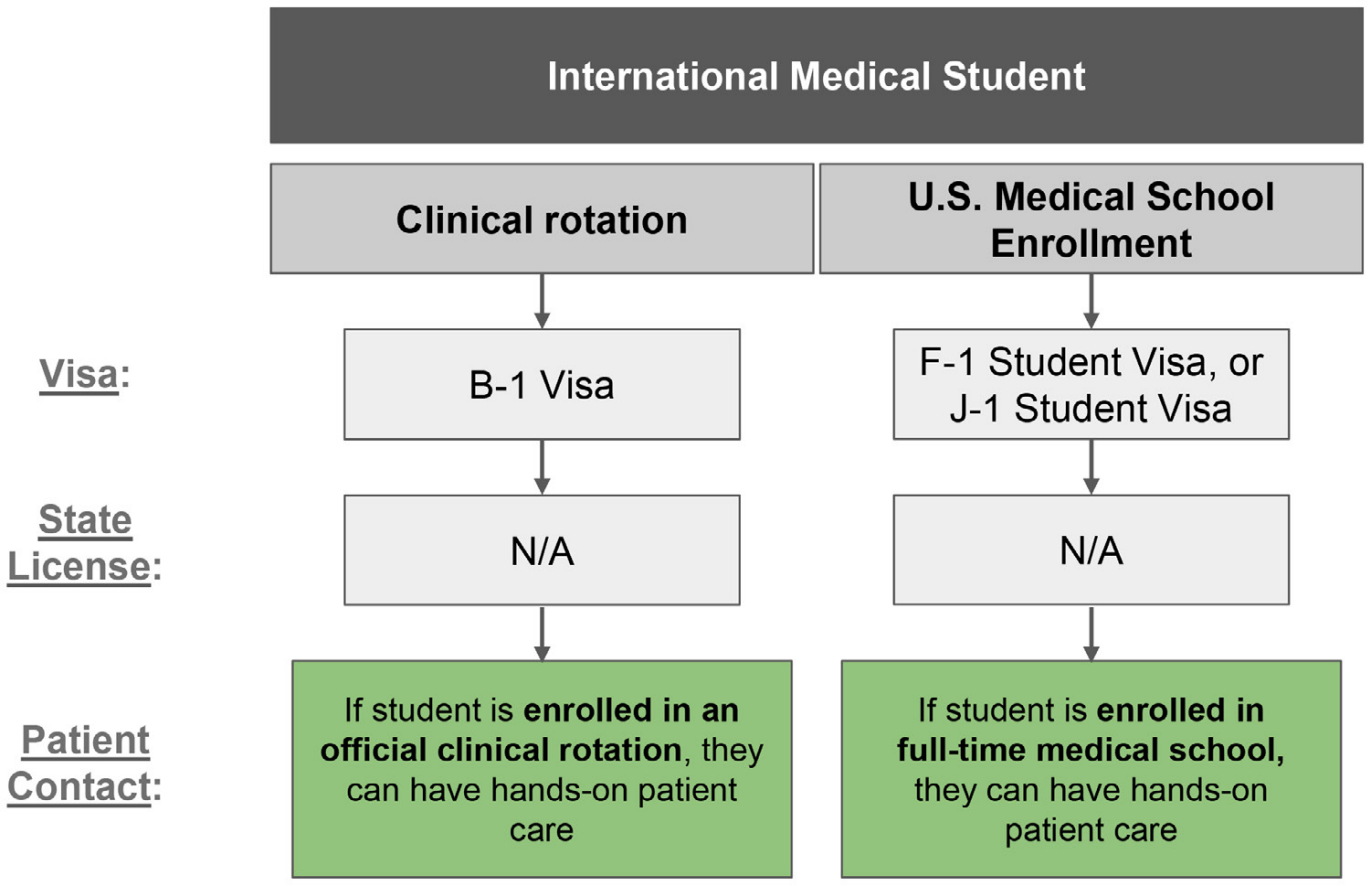
Illustrating pathways for international medical students, arranged side by side. Each pathway starts with visa type, followed by an arrow to state medical license eligibility, and then an arrow to whether patient contact is permitted.

**Figure 2:**
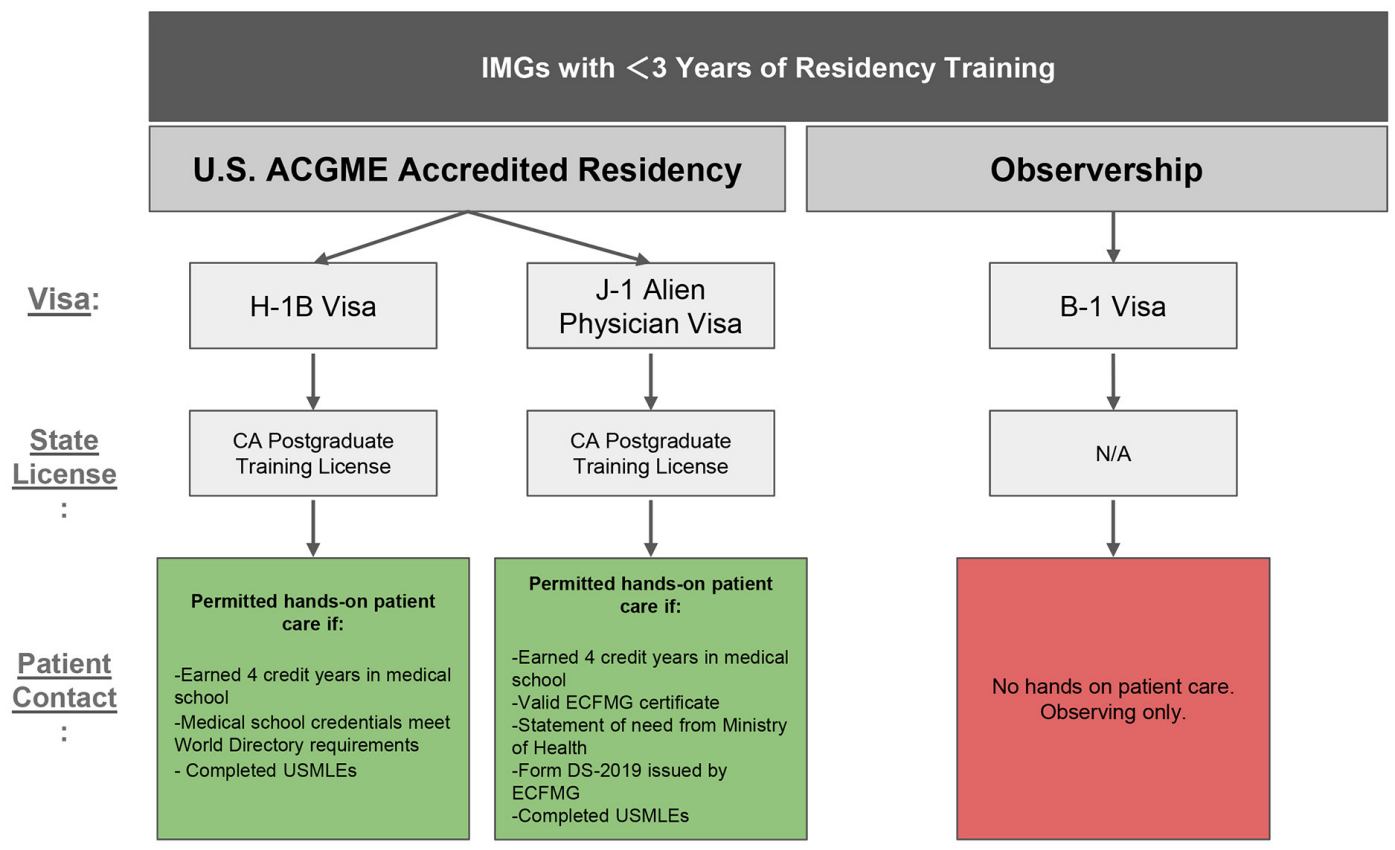
Illustrating pathways for international medical graduates with less than 3 years of residency training, arranged side by side. Each pathway starts with visa type, followed by an arrow to state medical license eligibility, and then an arrow to whether patient contact is permitted. **Abbreviations:** ACGME, Accreditation Council for Graduate Medical Education; IMG, international medical graduate; ECFMG, Educational Commission for Foreign Medical Graduates; USMLEs, United States Medical Licensing Examinations.

**Figure 3:**
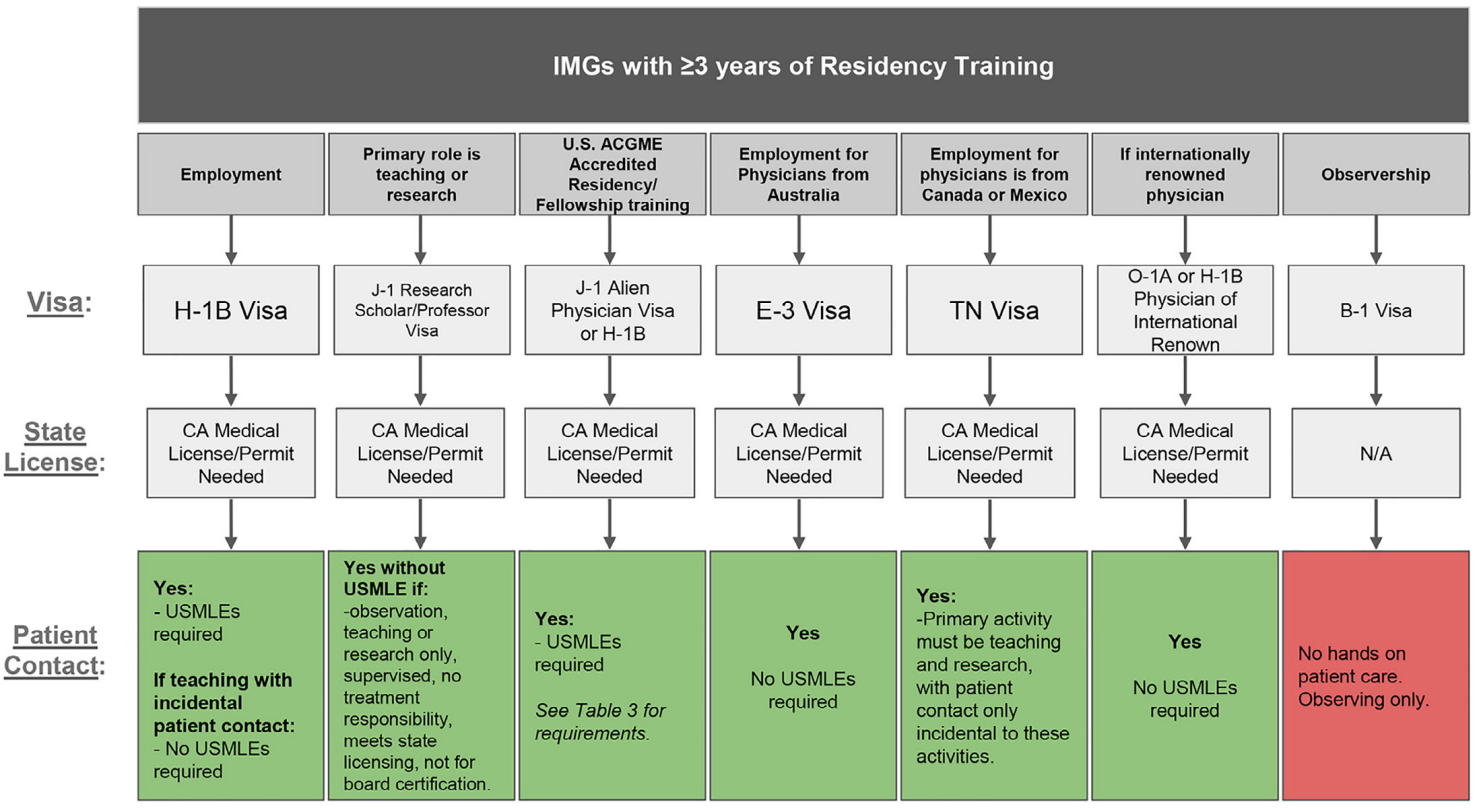
Illustrating pathways for international medical graduates with greater than or equal to 3 years of residency training. Each pathway starts with visa type, followed by an arrow to state medical license eligibility, and then an arrow to whether patient contact is permitted. **Abbreviations:** ACGME, Accreditation Council for Graduate Medical Education; USMLEs, United States Medical Licensing Examinations.

Several key strategies and resources have been identified to support the success of global health exchange programs. International medical students may undertake hands-on clinical experiences in the US under a B-1 visa. Navigating visa pathways for IMGs is considerably more complex, though viable pathways exist (e.g., J-1, H-1B, E-3, TN, O-1A), each designated for specific purposes. See Figures 1–3 for an overview of visa pathways. Additionally, California’s (Medical License) Special Permits allow IMGs to engage in hands-on clinical work under supervision.

Though funding remains a challenge, some programs have developed innovative solutions. UCLA’s David Geffen School of Medicine restructured its Travel Grant into the Global Health Collaboration Award to support bilateral partnerships. Instead of the prior focus on allocating funds to UCLA personnel to pursue training or research abroad, the new award specifically supports bilateral clinical, educational, and training initiatives with international partners, including specialty training, workshops, education initiatives, quality improvement projects, observerships, and research. The proposals must be developed in close partnership with international collaborators and be driven by their specific objectives [18]. In addition to redefining grant priorities, other avenues include reaching out to university-affiliated credit unions, which can help visitors open bank accounts, and financial coordination with schools of medicine can prevent unintended tax burdens on LMIC participants.

Administrative success often depends on collaboration and resource-sharing between experienced global health programs. Clear and compliant sponsorship letters, required by the Medical Board of California for Special Permit applications and by the US Department of State for visa processing, are essential to facilitating these exchanges. Additionally, well-structured training affiliation agreements (TAAs) play a critical role in supporting the process. UCSF’s TAAs with Ross University (Barbados) and St. George’s University (Grenada), for example, enabled annual clinical rotations at UCSF Fresno, though many TAAs remain unidirectional and short-term. Building long-term, reciprocal agreements is a pressing need.

At the individual level, tailored support and structured orientation can ease transitions. Programs like UCSD’s Visiting Clinical Fellowship customize experiences by balancing research, teaching, observation, and patient care based on each visitor’s background.

See Table 1 for a summary of institutional barriers and enablers.

**Table 1:** Barriers and Enablers to short-term clinical training opportunities.

BARRIERS
**Immigration / Visas**
Visa approval is not guaranteed
Expensive and time-consuming visa application process (recommended to start 1 year in advance)
*For IMGs:* visa processes are more complex due to the requirement of a CA Medical License or Special Permit for clinical interactions
**CA Medical License**
Need US SSN or Individual Taxpayer Identification Number
Need fingerprint clearances from both the DOJ and the FBI
California’s Special Permits can be challenging to use due to their limits within a given visa category
Variability in medical licensing requirements and recognition of qualifications across countries
**Funding**
Salary/stipend considerations
Travel and transportation costs
Sustainability of funding for training programs
Cost of living/housing in the host country
Restrictions imposed by donors on how funds can be used (e.g., funds designated for research supplies only)
Institutional funding limited to supporting US trainee activities
Limited access and cost of malpractice insurance, general liability insurance, health insurance, and medical evacuation insurance
May need passports and proof of address for banking services
Lack of US credit history makes it difficult to obtain credit cards
**US Institutional Capacity**
Concern over displacing local students and residents from training opportunities
Tailoring clinical supervision according to individual’s needs
Extensive paperwork: emails, letters, announcements, and coordination with multiple departments
Setting up review committees and rubrics for applications
Arranging clinical visits across multiple hospitals with different policies
Supporting visitors in adjusting to cultural differences
Established programs are easier to manage, while new programs face greater hurdles
**Visitor Logistics**
Adjust to new environments, including transportation, food, language, community, and spiritual needs
Family considerations, including whether spouses or children can be supported by the program to come to the US
Vaccination status and health clearances
**LMIC Institution Capacity**
Some LMIC institutions can partially or fully fund their trainees, which can be an incentive for US institutions but may limit partnerships to better-resourced partners
Determining the training experience desired by LMIC partners vs. what the host institution can offer
Differences in medical curricula and years of training across institutions (e.g., the experience of a 4th-year medical student varies)
LMIC institutions lack the capacity to cover the workload of the traveling IMG
**Political Climate**
Support for global health programs can wax and wane with changes in leadership and political priorities
**ENABLERS**
**Immigration / Visas**
International medical students can come to the US for hands-on clinical experience using a B-1 visa
Small number of existing visa pathways for IMGs (e.g., J-1, H-1B, E-3, TN, O-1A) for specific use cases. See Figures 1–3 for visa pathways.
**CA Medical License**
California’s Special Permits enable IMGs to interact with patients in clinical settings
**Funding**
Expand US university-affiliated grant criteria to allow funding for international partners
Structure program descriptions to demonstrate institutional value, thereby allowing visiting trainee expenses to be classified as business expenses
Revenue generation for global health programs can be achieved through institutional fees, grants, corporate sponsorships, clinical services, alumni donations, and other innovative partnerships
Open bank accounts for international medical students and IMGs via university-affiliated credit unions
**Administrative Support**
Collaborate with global health programs that have successfully managed international exchanges
Intentional and compliant sponsorship letters for the Medical Board of CA and visa support letters for the US State Department
Establish institutional training affiliation agreements to facilitate bidirectional global health partnerships
**Personal Considerations**
Provide comprehensive orientation programs to make international medical students and IMGs feel welcome
Offer city exploration programs to help international medical students and IMGs acclimate to their new environment
**Variability in Needs of LMIC Institution**
Programs that prioritize the needs of LMIC partners in the design of training programs
**Training Level**
IMGs have more experience than international medical students facilitating easier transition

**Abbreviations:** CA, California; DOJ, Department of Justice; FBI, Federal Bureau of Investigation; IMG, international medical graduate; LMIC, low- and middle-income country; SSN, Social Security Number.

### Navigating california licensure and special permits

A core requirement for IMGs pursuing Accreditation Council for Graduate Medical Education (ACGME) accredited residency or fellowship programs is the Postgraduate Training License (PTL), issued by the Medical Board of California [19]. It permits practice only within the program’s approved sites for the duration of training, and is not available for non-ACGME training.

For non-ACGME clinical training programs, Special Permits authorized by the Medical Board of California enable internationally trained physicians not yet eligible for full licensure to practice in supervised, defined settings. These include the Special Faculty Permit (BPC §2168) for academically eminent physicians and Special Permits (BPC §§2111, 2112, 2113) for postgraduate study, fellowships, or faculty roles [20–23].

Despite their potential, Special Permits remain underutilized for short-term training opportunities and tend to favor applicants from high-income countries. Requirements like a US SSN or ITIN, recognized school credentials, and academic titles limit LMIC participation. Each permit has strict eligibility rules—sponsorship letters, residency verification, fingerprint clearance, and practice restrictions. Visa incompatibilities further limit their use, hindering many California global health programs from leveraging these permits effectively.

See Table 2 for a detailed summary of the CA Special Permits.

**Table 2:** CA Special Permits.

NAME OF PERMIT	# ISSUED IN 2024 IN CA	PURPOSE OF LICENSE	TIME RESTRICTIONS	LOCATION RESTRICTIONS	QUALIFICATIONS
**Special Faculty Permit 2168**	6	For IMG physicians sponsored by the dean of a California School of Medicine, recognized as academically eminent, who do not meet licensure requirements for unrestricted CA medical license.	None mentioned.	Practice is restricted to the sponsoring medical school and its affiliated institutions.	Full-time appointment at the level of full professor (or equivalent) in a tenure track at a California medical school approved by the Board. Graduated from a medical school recognized by the Medical Board of California. Completed at least 3 years of basic postgraduate residency training. Hold a license to practice in another state, Canadian province, foreign country, or jurisdiction. Not held a position under Section 2113 for 2 years prior to application. Have a US SSN or ITIN prior to issuance of a permit. Fingerprint clearances from DOJ and FBI prior to issuance of a permit.
**Special Programs 2111**	21	For IMGs seeking postgraduate medical school study in California under the title of “visiting fellow.”	3 years, renewable annually.	Restricted to professional activities within the appointed California medical school.	Graduated from a medical school recognized by the Medical Board of California. Completed at least 3 years of basic postgraduate residency training. Have a US SSN or ITIN prior to issuance of a permit. Fingerprint clearances from DOJ and FBI prior to issuance of a permit.
**Special Programs 2112**	1	For IMGs participating in a fellowship program in a specialty or subspecialty field.	Issued for 1 year, renewable up to four times.	Restricted to hospitals in California approved by the Joint Commission.	Graduated from a medical school recognized by the Medical Board of California. Have a US SSN or ITIN prior to issuance of a permit. Fingerprint clearances from DOJ and FBI prior to issuance of a permit.
**Special Programs 2113**	7	For IMG physicians with a full-time faculty position, allowing practice incidental to their faculty duties.	1 year, renewable twice.	Restricted to within the host institution.	Graduated from a medical school recognized by the Medical Board of California. If from a non-US/Canadian school: at least 4 years of licensure, practice in an approved US facility, or a combination. Have a US SSN or ITIN prior to issuance of a permit. Fingerprint clearances from DOJ and FBI prior to issuance of a permit.

**Abbreviations:** IMG, international medical graduate; CA, California; SSN, Social Security Number; ITIN, Individual Taxpayer Identification Number; DOJ, Department of Justice; FBI, Federal Bureau of Investigation.

### Current pathways for clinical global health exchanges at california AMCs

#### International medical students

Several pathways exist for international medical students to train in California (Figure 1). Those enrolled full-time in US medical schools typically use F-1 or J-1 visas and do not require a license to perform clinical care defined within their coursework (e.g., patient histories, physical exams).

For short-term clinical training, international medical students may enter on a self-sponsored B-1 visa to complete a clinical rotation approved by the medical school dean. Under this visa, students are allowed to engage in hands-on patient care under the supervision of a licensed physician. Compared to IMGs, international medical students face fewer regulatory barriers to clinical participation [24].

Successful implementations of this pathway for international medical students include programs at Stanford and UCLA. Stanford’s International Visiting Student Program makes its electives broadly accessible to international students enrolled in their final year of medical school [25]. UCLA and UCSF Fresno allow clinical rotations for students from international medical schools that have established affiliation agreements with the institution [26, 27]. These TAAs are particularly valuable because they can be structured to promote bidirectional and equitable exchanges, creating reciprocal opportunities for both UCLA students and their international counterparts.

While the pathway for international medical students to participate in short-term, hands-on clinical experiences is well established, there remain limited opportunities across California. For example, the AAMC VSLO program lists more than 900 clinical electives statewide, but many California medical schools do not offer these opportunities to international medical students [28–32]. Moving forward, California medical schools should prioritize developing and sustaining bilateral, equitable exchange programs that strengthen global academic partnerships and expand access to clinical training.

#### IMGs with less than 3 years of residency training

Figure 2 shows the limited pathways for IMGs with under 3 years of residency training. IMGs may enter full-time ACGME residency programs on J-1 or H-1B visas paired with a California PTL. The J-1 visa requires ECFMG certification, USMLE Step 1 and 2 passage, a home country statement of need, and a CA license or permit, plus a 2-year return home post-training [33–35]. The H-1B visa, employer-sponsored, allows unrestricted clinical care with USMLE Steps 1–3, graduation from an ECFMG-recognized school, and a CA license or permit [33, 36]. However, recent federal policy shifts have raised the H-1B application fee from roughly $2,000–$5,000 to $100,000, a change that could significantly limit institutional use of this pathway unless physician exemptions are clearly defined and applied [16, 17]. No visa currently exists for short-term, hands-on clinical training for IMGs. Once an international medical student graduates, the B-1 visa only permits observerships without direct patient care [24]. This visa discrepancy poses a major barrier to IMG clinical training.

Although the California Special Permit (2112) allows supervised patient care for graduates of recognized international medical schools who have a US SSN/ITIN and fingerprint clearance, it remains rarely utilized. With appropriate visa pairing, this underutilized pathway could be further explored to support short-term training for IMGs with less than 3 years of residency training.

#### IMGs with at least 3 years of residency training

Figure 3 shows the pathways identified for IMGs with at least 3 years of residency training from any country. For employment in full-time ACGME residency or fellowship programs, IMGs may use the J-1 Alien Physician or H-1B visas as above, but apply for the 2111 California Special Permit, which allows patient interaction. This 2111 permit requires graduation from a Medical Board of California-approved school, 3 years of residency, a US SSN or ITIN, and DOJ/FBI fingerprint clearance.

Again, IMGs seeking short-term training opportunities cannot use the B-1 visa for hands-on clinical work, so many programs default to offering observerships, which exclude patient contact [24]. Several non-ACGME pathways exist for short-term clinical training, though these “short-term” programs typically last 1 year, similar to fellowship years. One such non-ACGME option hires experienced physicians as clinical instructors or visiting professors on the H-1B visa with the 2113 CA Special Permit, allowing “incidental” patient care related to teaching duties. This pathway is complex, requiring graduation from a Medical Board of California-approved school, 3 years’ residency, DOJ/FBI clearance, and a US SSN or ITIN—which can only be requested two weeks after arrival, further causing delays. Again, recent federal changes to H-1B costs add another layer of uncertainty, though exemptions for physicians may apply. The E-3 and TN visas may also be used for Australian, Mexican, or Canadian citizens, respectively, with the 2113 CA Special Permit [37, 38].

Another option is the J-1 Research Scholar or Professor visa with the 2111 Special Permit, which allows non-clinical roles that do not require passing the USMLE exams. According to 22 CFR 62.27(c), the program’s responsible officer must verify that no patient care is provided. Alternatively, the medical school dean must certify the “Five Points Memo,” which ensures that the role involves observation, consultation, teaching, or research; only supervised incidental patient contact; no final responsibility for patient care; compliance with state licensing requirements; and that the experience does not count toward specialty board certification [39]. UCLA, UC Irvine, and Stanford have historically defined “incidental patient contact” as under 20% of program time, but there is no guidance from USCIS [21, 40, 41]. The lack of clarity around what constitutes a “non-clinical” exchange in the context of this Five-Points Memo leaves room for interpretation. Clearer guidance from the State Department could expand the use of this model for short-term clinical exchanges without requiring USMLEs.

Additional visas include H-1B for Physicians of International Renown and the O-1A visa for extraordinary ability, both requiring state licensure but not USMLEs [42, 43]. Both options are inaccessible to the majority of LMIC physicians who lack the credentials needed for these pathways, making them impractical for broader use.

### Recommendations for equitable clinical training programs for international medical students and IMGs

#### Commit to reciprocity and institutional support

California AMCs must prioritize equitable global partnerships by treating the inclusion of international medical students and IMGs as mission-critical. Commitment to addressing the financial, administrative, cultural, and political barriers can further enable meaningful training opportunities. Even small commitments, such as hosting 1–2 international medical students or IMGs annually per institution, could collectively generate a significant impact.

A key step toward reciprocity is allocating funding to support inbound international students and trainees proportional to outbound US students and trainees. Limited resources can launch impactful programs, and fundraising partnerships with nonprofits and industry can supplement budgets. Formalizing TAAs is vital but often complex. Streamlining these processes and providing dedicated institutional resources, such as administrative support to handle visa and licensing issues, is critical. Grant opportunities targeting LMIC collaborators can further strengthen bilateral partnerships.

Importantly, LMIC partners, who have too often been excluded from shaping these efforts, should lead in defining exchange models. While CA AMCs may prioritize sending students and residents, LMIC institutions may prefer training junior or mid-career faculty who are positioned to drive long-term impact in local systems.

#### Reform visa and licensing policies

Visa and licensing policies require significant reform to expand short-term opportunities for IMGs. International medical students can use the B-1 visa for clinical rotations, and this pathway should be more frequently utilized to promote hands-on clinical training. For IMGs, long-term advocacy efforts should focus on developing a new J-1 visa category tailored to short-term clinical exchanges. One such example is the work of the Coalition for Building Reciprocal Initiatives for Global Health Training (Coalition BRIGHT), which has been working to gather support for a federal J-1 visa amendment proposal [44]. In the short term, advocacy efforts should prioritize a physician exemption to the recent H-1B executive order, which could otherwise drastically limit IMG training opportunities that utilize the H-1B visa.

Other avenues for progress include updating US Department of State guidelines to clarify “incidental” patient care under J-1 Research Scholar or Professor and H-1B visas, and solidifying existing pathways for IMGs. Incidental patient care should be defined as supervised clinical interactions where the individual does not have final responsibility for the diagnosis or treatment of patients and is in compliance with state licensing requirements. Creating a national framework for state medical boards to issue temporary short-term training licenses, like those for US residents, could simplify processes for institutions and IMGs. A full overview of visa options for short-term clinical training is presented in Table 3.

**Table 3:** Overview of visa pathways permitting hands-on clinical training for international medical students and IMGs.

	STUDENT AND EXCHANGE VISITOR			
VISA CATEGORY	F-1 STUDENT	J-1 STUDENT DEGREE	J-1 ALIEN PHYSICIAN	J-1 RESEARCH SCHOLAR/PROFESSOR
**Visa Purpose**	Full-time education	Full-time education, bachelors, masters, PhD, doctorate	Residency and Fellowship Training	**Research Scholar:** Focused on conducting research or consulting at research institutions **Professor:** Primarily teaching, lecturing, or consulting at accredited academic institutions
**Medical Training/ Employment Use**	US Medical School	US Medical School	ACGME-accredited residency, fellowship, and non-standard training programs	Primary purpose is participation in non-clinical programs for observation, consultation, teaching, and research
**Possibility for Clinical Contact**	Yes, if patient contact is required for the curriculum	Yes, if needed for degree completion Patient contact does not require approval if the university can issue Form DS-2019 for J-1 students	Yes, within ACGME limitations	Yes, if: Program focuses on observation, consultation, teaching, or research Patient contact supervised by a licensed US physician No final responsibility for diagnosis or treatment Compliant with State licensing rules Experience does not count toward board certification
**How visa *cannot* be used**	GME Residency/Fellowship Physician employment	GME Residency/Fellowship Physician employment	Clinical research outside approved GME programs Clinical fellowships/activities by non-ACGME programs No employment beyond approved training program (i.e. moonlighting)	GME Residency/Fellowship No employment beyond approved training program (i.e. moonlighting)
**Required before applying for US Visa**	Completion of all standard educational prerequisites Official school acceptance Proof of sufficient financial resources to cover attendance costs Form I-20 issued by the sponsoring university	Completion of all standard educational prerequisites Official school acceptance Proof of sufficient financial resources to cover attendance costs Majority of funding must come from external, non-personal, or family sources Form DS-2019 issued by the sponsoring university	Valid ECFMG certificate Completion of USMLE Steps 1, 2CK, 2CS Statement of need from the Ministry of Health Official GME residency/fellowship contract Form DS-2019 issued by ECFMG	Minimum bachelor’s degree with relevant research or teaching experience Offer letter Certification from medical school dean regarding incidental patient contact
**Duration of US Stay**	Normal full-time study (typically 4 years) 1–3 years of optional practical training possible post completion	Normal full-time study (typically 4 years) Up to 36 months of academic training possible post-graduation	Time to complete US board certification in the specialty (up to 7 years)	Up to 5 years

**Table:** 

	SELF-SPONSORED	
VISA CATEGORY	B-1	VISA WAIVER PROGRAM (AS WAIVER BUSINESS)
**Visa Purpose**	Business Visit	Business Visit
**Medical Training/Employment Use**	Business consultation Conference attendance Interview Non-clinical observership Short-term medical clerkship for international students	Business consultation Conference attendance Interview Non-clinical observership Short-term medical clerkship for international students
**Possibility for Clinical Contact**	Yes, only for medical students on approved clerkship rotation at a US medical school	Yes, only for medical students on approved clerkship rotation at a US medical school
**How visa *cannot* be used**	GME Residency/Fellowship Research Employment Any compensated activity or service	GME Residency/Fellowship Research Employment Any activity or service for compensation
**Required before applying for US Visa**	Travel plan/itinerary Employer letter or invitation/registration proof for business, educational, or commercial event	Travel plan/itinerary Employer letter or invitation/registration proof for business, educational, or commercial event
**Duration of US Stay**	Typically 6 months	90 days

**Table:** 

	EMPLOYER-SPONSORED				
VISA CATEGORY	H-1B	H-1B PHYSICIAN OF INTERNATIONAL RENOWN	O-1A	E3	TN
**Visa Purpose**	Specialty occupation, sponsored by US employer	Clinical physicians seeking H-1B nonimmigrant visas	Extraordinary ability. Individuals that have risen to international acclaim in their field	For citizens of Australia to work in specialty occupations with US employer sponsorship	For citizens of Canada or Mexico to work in identified occupations in the US
**Medical Training/ Employment Use**	GME residency and fellowship programs Postdoctoral trainees Physician employment Faculty in non-ACGME programs	Physician employment Faculty in non-ACGME programs	Work clinically as a physician, must also possess state license	GME residency and fellowship programs Postdoctoral trainees Physician employment Faculty in non-ACGME programs	Allows qualified Canadian and Mexican citizens temporary entry into the US for professional business activities
**Possibility for Clinical Contact**	Clinical work requires USMLEs and approved CA medical license/permit Teaching roles with incidental patient contact require a CA medical license/permit, but not USMLEs	Yes, if demonstrates national or international renown per USCIS evidence list	Yes, does not require USMLEs	Yes, with state medical license/permit	Only for teaching/research; patient interaction must be incidental
**How visa *cannot* be used**	Unpaid work	Unpaid work	Physicians who have not met the requirements for O-1	Unpaid work	Primary purpose of patient care
**Required before applying for US Visa**	Applicant must have 4 credit years or an MD equivalent Medical school must meet requirements via https://www.wdoms.org/ For clinical work: USMLE Steps 1, 2CK, 2CS, and 3 required For teaching: No USMLE if clinical contact is “incidental” per Department of Homeland Security Approved state medical license for the work	Approved state medical license appropriate for the work activity No USMLEs if they demonstrate qualification under the national or international renown standard	Sponsoring employers must show that O-1 visa recipients have a major internationally recognized prize or meet at least three USCIS eligibility requirements	Must be an Australian national Must have an approved Labor Condition Application from the Department of Labor Employment must qualify as a “specialty occupation” Must have the necessary academic qualifications Must intend to leave the US when E-3 status ends Must have a state license	Proof of Canadian or Mexican citizenship MD or Doctor en Medicina; State/Provisional license (post-entry) Employer letter (role, purpose, duration, qualifications) Credentials evaluation (if applicable) TN employer files Form I-129, then apply for TN entry Canadian: Enter US in TN status at port of entry Mexican: Apply for TN visa at US embassy/consulate
**Duration of US Stay**	Maximum 6 years, with extensions Issued in 3-year increments	Maximum 6 years, with extensions Issued in 3-year increments	Initial stay of 3 years, with annual 1-year extensions	Up to 2 years per extension	3 years initially, extendable in 3-year increments

**Abbreviations:** ACGME, Accreditation Council for Graduate Medical Education; CA, California; DOJ, Department of Justice; DS-2019, Certificate of Eligibility for Exchange Visitor (J-1) Status; ECFMG, Educational Commission for Foreign Medical Graduates; FBI, Federal Bureau of Investigation; GME, Graduate Medical Education; IMG, international medical graduate; I-20, Certificate of Eligibility for Nonimmigrant Student Status; MD, Doctor of Medicine; SSN, Social Security Number; USCIS, US Citizenship and Immigration Services; USMLE, United States Medical Licensing Examination; VWP, Visa Waiver Program.

At the state level, more support is needed for IMGs with less than 3 years of residency. The 2112 California Special Permit, allowing clinical interaction for IMGs with less than 3 years of residency training, is rarely used. Proper visa alignment could unlock its potential. California should also expand Special Permits to include a new category for supervised clinical care during short-term rotations, including graduates from international schools who are currently not recognized by the Medical Board of California.

#### Increase awareness and utilization of special permits

Increasing awareness of California’s Special Permits through workshops, webinars, and campaigns can educate IMGs, medical schools, and global health programs and immediately expand opportunities. International students’ offices can guide applicants as clinical training programs are established.

California AMCs could incentivize bidirectional programs with grants or recognition, encouraging permit use and strengthening global health efforts. Global and local consortia, such as the Bay Area Global Health Alliance or the Consortium of Universities for Global Health, are potential forums to bring California institutions together and serve as a model for future advocacy groups.

See Table 4 for a summary of recommendations.

**Table 4:** Summary of recommendations for equitable short-term clinical training programs for international medical students and IMGs.

STAKEHOLDER	CURRENT STATUS	PROPOSED REFORMS
**International Medical Students**	Limited clinical rotation opportunities	Advocate for California medical schools to expand visiting student programs to include international medical students Encourage US medical institutions to dedicate funding and efforts to bring LMIC students to the US when creating global health exchange programs
**IMGs with < 3 Years of Residency**	Severely restricted clinical practice; CA 2112 Special Permit exists but rarely used (only one permit issued in 2024)	Increase awareness and use of California’s Special Permits for IMGs through targeted outreach, partnerships with global health networks, and institutional incentives to expand bidirectional clinical training programs Expand California’s Special Permits to include a new category allowing supervised clinical interactions for short-term global health exchanges Within this new Special Permit category, include graduates from international medical schools not currently recognized by the Medical Board of California to increase IMG participation from LMICs
**IMGs with > 3 Years of Residency**	Pathways exist, but are complex; limited short-term opportunities due to visa and licensing restrictions	Advocate for a new J-1 visa category for short-term clinical exchanges Propose regulatory changes to permit supervised “incidental” patient care under J-1 Research Scholar/Professor and H-1B visas, defined as supervised clinical interactions without final responsibility for patient care and in compliance with state licensing laws Establish a universal framework for temporary short-term training licenses across state medical boards
**CA Academic Medical Centers**	US institutions focus primarily on outbound programs; funding and administrative capacity for inbound exchanges are often lacking	Designate global health exchanges as a mission priority Dedicate budget and staff for inbound IMGs and students Commit to even small-scale programs (1–2 trainees/year) Pursue external fundraising where internal funds are limited Simplify legal agreements for partnerships Create grant opportunities specifically for LMIC partners
**LMIC Partner Institutions**	Programs are often designed primarily by US institutions, with LMIC partners in supporting roles	Ensure LMIC partners lead design of exchanges aligned with local priorities. Focus exchanges on building LMIC health system capacity Avoid programs that incentivize permanent migration (“brain drain”)

**Abbreviations:** CA, California; IMG, international medical graduate; LMIC, low- and middle-income country.

## Conclusion

There is a longstanding need to address inequities in clinical training for international medical students and IMGs at CA AMCs. While barriers are complex, institutions can adopt key enablers to overcome them. Through advocacy at federal, state, and institutional levels, including the creation of new visa categories tailored to short-term clinical exchanges, and by promoting inclusive, sustainable exchange programs, California can leverage its robust training infrastructure to lead in global health equity. These efforts align with California’s health goals by recognizing the deep interconnection between local and global health: promoting diversity, cultural humility, and broader system resilience that directly benefit care for California’s increasingly diverse communities.
